# Supplementary material for: Content-rich biological network constructed by mining PubMed abstracts
Source: BMC Bioinformatics. 2004 Oct 8;5:147. doi: 10.1186/1471-2105-5-147 (PMC528731; doi:10.1186/1471-2105-5-147)
Supplement: Additional File 2 — The original results of the above study (non-essential files are deleted to keep the file size under the limit set by BMC bioinformatics). [file 1471-2105-5-147-S2.bz2 › chilibotAdditionalFile2/dip05/744ID10419942E18123/html/TOLQ_TOLR.html]

 


 **TOLQ** and **TOLR** 
  
Found 36 abstracts in PubMed, retrieved 05.  
 

 What does Google say? 
 PDF only 
| .edu only 

---

**Interactive relationship** (e.g. stimulation, inhibition, etc)

**Non-interactive relationship** (e.g. studied together, co-existance, homology, etc.)

- The Tol Pal system of Escherichia coli is composed of the YbgC,  **TolQ** , TolA,  **TolR** , TolB, Pal and YbgF proteins.  Ref: 11994151 Mol Microbiol, 2002
- Analysis of polar mutations, reverse transcriptase PCR assays, and transcriptional fusion constructs with a promoterless lacZ gene revealed that the genes are arranged in two operons orf1  **tolQ**   **tolR**  tolA tolB and oprL orf2.  Ref: 12486055 J Bacteriol, 2003
- In Pseudomonas putida, the seven genes, orf1,  **tolQ** ,  **tolR** , tolA, tolB, oprL, and orf2, which encode the proteins of this complex, are clustered in a 5.8 kb region of chromosomal DNA.  Ref: 12486055 J Bacteriol, 2003
- Six of these new genes encode different homologues of the  **TolR** , TolB, and  **TolQ**  transport proteins, suggesting that adventurous motility is dependent on biopolymer transport.  Ref: 12828649 Mol Microbiol, 2003
- Bacteria carrying tolA,  **tolQ**  and  **tolR**  mutations kept their colicin insensitivity as spheroplasts, just as the resistant ones.  Ref: 12094727 Folia Microbiol Praha , 2002
- It was shown that the TolA,  **TolQ** , and  **TolR**  proteins play a critical role in the translocation of group A colicins and filamentous bacteriophage minor coat proteins g3p.  Ref: 11851406 Biochemistry, 2002
